# Targeting neutrophil-driven inflammation in adult-onset still’s disease: molecular insights from gene expression profiles

**DOI:** 10.1186/s13075-025-03598-5

**Published:** 2025-07-01

**Authors:** In-Woon Baek, Hyoun-Ah Kim, Kyung-Su Park, Ki-Jo Kim

**Affiliations:** 1 Division of Rheumatology, Department of Internal Medicine, College of Medicine, Ewha Womans University, Seoul, Republic of Korea; 2 Department of Rheumatology, Ajou University School of Medicine, Suwon, Republic of Korea; 3 Division of Rheumatology, Department of Internal Medicine, College of Medicine, St. Vincent’s Hospital, The Catholic University of Korea, 93 Jungbu-daero, Paldal-gu, Suwon, 16247 Gyeonggi-do Republic of Korea

**Keywords:** Adult-onset still’s disease, Neutrophil activation, Interleukin-1, Chemokine receptors

## Abstract

**Background:**

The rarity and heterogeneity of adult-onset Still’s disease (AOSD) pose significant challenges in understanding its precise pathogenic mechanisms, developing effective treatment options, and establishing therapeutic strategies. A comprehensive analysis of gene expression profiles could help to bridge the knowledge gaps in those areas.

**Methods:**

A blood transcriptomic dataset comprising 31 patients with AOSD and 22 healthy controls was fetched. Cellular and molecular features were identified by analyzing differentially expressed genes (DEGs) and functional enrichment. Optimal molecular targets for neutrophil activation were identified using kernel-based diffusion scoring techniques.

**Results:**

Blood molecular signatures indicate that neutrophil degranulation is the most enriched pathological process in AOSD. Neutrophil degranulation correlated significantly with the expression of Fcγ receptors, IL-1 receptors, and chemokine receptors and their signaling activities. IL-1 inhibitors and IL-6 inhibitors did not exhibit a diffusion score favorable for directly deactivating neutrophil degranulation, but agents targeting CXCR1/CXCR2, C5AR1, neutrophil elastase, SRC, and SYK demonstrated significant diffusion scores for neutrophil degranulation. In particular, CXCR1, CXCR2, and C5AR1 were the DEGs predominantly expressed in neutrophils and closely associated with neutrophil degranulation in a context-specific functional analysis.

**Conclusions:**

Neutrophil activation is a key pathological module in AOSD. Therapeutic approaches aimed at neutrophils could offer a promising opportunity to regulate the inflammatory response in AOSD.

**Supplementary Information:**

The online version contains supplementary material available at 10.1186/s13075-025-03598-5.

## Introduction

Adult-onset Still’s disease (AOSD) is a systemic autoinflammatory disease of unknown etiology that is clinically characterized by spiking fever, arthritis, evanescent rash, and hyperferritinemia [1, 2]. AOSD shares many clinical manifestations and mechanistic features with systemic juvenile idiopathic arthritis (sJIA) and it is considered to be an adult form of the Still’s disease spectrum [3].

AOSD typically affects young adults; however, its incidence is very low. There is significant heterogeneity in clinical presentation, and no specific diagnostic test has been developed. Therefore, delays in the diagnosis of AOSD are common [1, 4]. Its clinical heterogeneity impedes the study and understanding of the disease and the development of optimal management strategies. Current therapeutic strategies primarily rely on clinical experience and expert opinions. For at least 30–40% of patients, the disease cannot be controlled using the traditional approaches [5, 6]. The introduction of targeted biologic agents developed to treat other chronic rheumatic diseases has significantly changed the treatment of AOSD. However, their efficacy is variable, and most of the relevant studies have been small-scale case series or had short-term follow-up or retrospective observational designs [2, 4, 7, 8]. As observed in multiple studies, AOSD is associated with significant morbidity and mortality and thus seriously affects the health-related quality of life of patients [1].

The current understanding of the disease mechanism of AOSD involves the activation of both innate and adaptive immunity, mediated by a pro-inflammatory cytokine storm [4, 8]. Cytokines, including interleukin (IL)-1, IL-6, IL-8, IL-17, IL-18, and tumor necrosis factor (TNF), contribute to an amplified inflammatory response. The activation and interactions of neutrophils and monocytes circulating in the blood are considered to be key factors in this process. Current treatment strategies are limited to non-selective anti-inflammatory and selective anti-cytokine agents, and no modality effectively target the underlying cellular components.

Blood transcriptome profiling has been used in various medical fields to define disease signatures and investigate mechanisms associated with, and potentially implicated in, disease pathogenesis [9]. Particularly for systemic inflammatory diseases that do not have unique target organ(s), such as AOSD, blood samples from patients are a valuable source for deriving a deeper understanding of the cellular and molecular alterations involved in the disease mechanism. For this study, we imported blood transcriptomic data obtained from patients with AOSD and used advanced bioinformatic tools to examine the cellular and molecular characteristics that orchestrate the inflammatory process. We investigated the key pathogenic processes and identified the molecules associated with those processes. Using a kernel-based network diffusion algorithm, we examined the limitations of current treatment strategies, and we propose promising therapeutic targets that might enable effective control of the key disease module driving the inflammatory mechanisms of AOSD.

## Methods

### Data collection

We imported blood transcriptomic datasets from GEO: GSE22098 for AOSD [10, 11] and GSE80060 for sJIA [12, 13]. Blood samples were collected 31 patients with AOSD (females = 15 (48.4%), age = mean 41 (interquartile range 27–54) years-old) and 22 corresponding healthy controls and from 104 patients with sJIA and 22 corresponding healthy controls (Supplementary Tables 1 and 2).

### Preprocessing of gene expression data

All analyses were conducted in R (version 4.5.0, The R Project for Statistical Computing, www.r-project.org). Using Affymetrix microarrays, the robust multi-array average (RMA) method was used for background correction, normalization, and probe set summarization [14]. The RMA uses quantile normalization, which equalizes the data distributions of the arrays and makes the samples completely comparable. We used RMA in the R package affy for the one-channel Affymetrix arrays. Illumina gene expression array data were preprocessed using the lumi package and normalized using the robust spline normalization method [15], which combines the features of quantile and loess normalization. Residual technical batch effects arising from the integration of multiple heterogeneous datasets were adjusted using the ComBat method [16], which uses either parametric or non-parametric empirical Bayes frameworks to adjust the data for batch effects. We adopted a combination of quantile normalization and the ComBat function for quality control because it has demonstrated better performance than other methods [17, 18]. Quality assurance and distribution bias were assessed using a principal component analysis. After preprocessing, systematic and dataset-specific biases were significantly reduced compared with before normalization and batch correction (Supplementary Fig. 1).

### Filtration of differentially expressed genes

Differentially expressed genes (DEGs) were identified using the limma package, which takes an empirical Bayes approach [19]. P-values were adjusted using the Benjamini-Hochberg method, and DEGs were defined as those with an adjusted P-value of less than 0.05 and an absolute fold change greater than 1.3.

### Gene-set and cell-type enrichment analyses

A functional enrichment analysis of the upregulated DEGs was conducted in Enrichr software, which uses adjusted *P*-values, odds ratios and the combined scores as the featured enrichment indices [20]. The enrichment results were visualized using the Enrichment Map format, in which nodes represent gene sets and weighted links between the nodes indicate an overlap score based on the number of genes shared between those two gene sets (Jaccard similarity coefficient) [21].

A gene-set enrichment analysis (GSEA) was used to identify a group of genes in a specified list of genes that are overrepresented compared with a background set of genes [22]. The normalized enrichment score (NES) was used to compare analysis results across gene sets by accounting for variations in gene set size and correlations between the sets and the expression dataset. Gene-set information about the signaling pathways and biological processes was obtained from the Kyoto Encyclopedia of Genes and Genomes, Gene Ontology (GO), and the Reactome database [23–25]. Single-sample enrichment scores for the gene sets were estimated with a gene-set variation analysis (GSVA) using the ‘gsva’ function from the R package GSVA [26]. Enriched processes were further evaluated through a blood transcriptome modular repertoire analysis [9, 27], which includes 382 transcriptome modules derived from co-expression patterns of blood genes. Information about the modules is available at https://ayllonbe.github.io/modulesV3/index.html. The module response is represented as the percentage of transcripts within a specific module that exhibits significant differential expression between study groups. xCell 2.0 software, an advanced cell type deconvolution method, was used to estimate the cell type-specific proportions and enrichments of molecular signatures [28].

### Interferon-stimulated genes scores

The interferon (IFN)-stimulated gene (ISG) scores were calculated using the AUCell package [29] and a list of ISGs was fetched from the Molecular Signatures database [30].

### Human protein-protein interaction network and identification of influential nodes

A human protein-protein interaction (PPI) network was constructed using the Human Interactome database [31]. Nodes and edges represent proteins and their functional or physical interactions, respectively. The network comprises 18,557 nodes and 482,017 edges. Hub nodes were determined using Kleinberg’s hub centrality scores [32].

### Network-based diffusion scoring

To quantify the leverage of a specific gene on a subnetwork through its links, we used label propagation and a network diffusion algorithm, which models heat flow from seed genes along the interactions in a PPI network [33]. diffuStats, an R package that provides a collection of graph kernels and diffusion scores, and a *z*-scaled Monte-Carlo method were used with default parameters to calculate a diffusion score for the nodes based on the pathway-specific gene sets. Information on the target molecules of drugs was retrieved from DrugBank (https://go.drugbank.com/) [34].

### Single cell RNA sequencing data analysis

Single cell RNA sequencing data of human blood cells including neutrophils was fetched from the GSE137540, which was used for study of neutrophil heterogeneity in homeostasis and infection [35]. This data included single-cell RNA sequencing of 29,909 single cells isolated from the blood of three healthy donors, which were processed using the Seurat package according to the standard protocol [36] and the clustered cell types were annotated by singleR package [37].

### Detection of the context-specific functions of individual genes

We used the geneCOCOA algorithm, which computes gene set-specific enrichment P values and differential scores between two conditions, such as disease and control, to identify context-specific changes in gene function [38]. This algorithm allows users to infer the putative functions of a gene-of-interest (GOI) based on the co-expression of the given gene and curated sets of functionally annotated genes.

### Statistical analysis

For continuous distributed data, between-group comparisons were performed using unpaired *t*-tests. Categorical or dichotomous variables were compared using the chi-squared test or Fisher’s exact test. Correlation analyses between two variables were carried out using Pearson’s method.

## Results

### Differential enrichment of AOSD-associated pathways

**Fig. 1:**
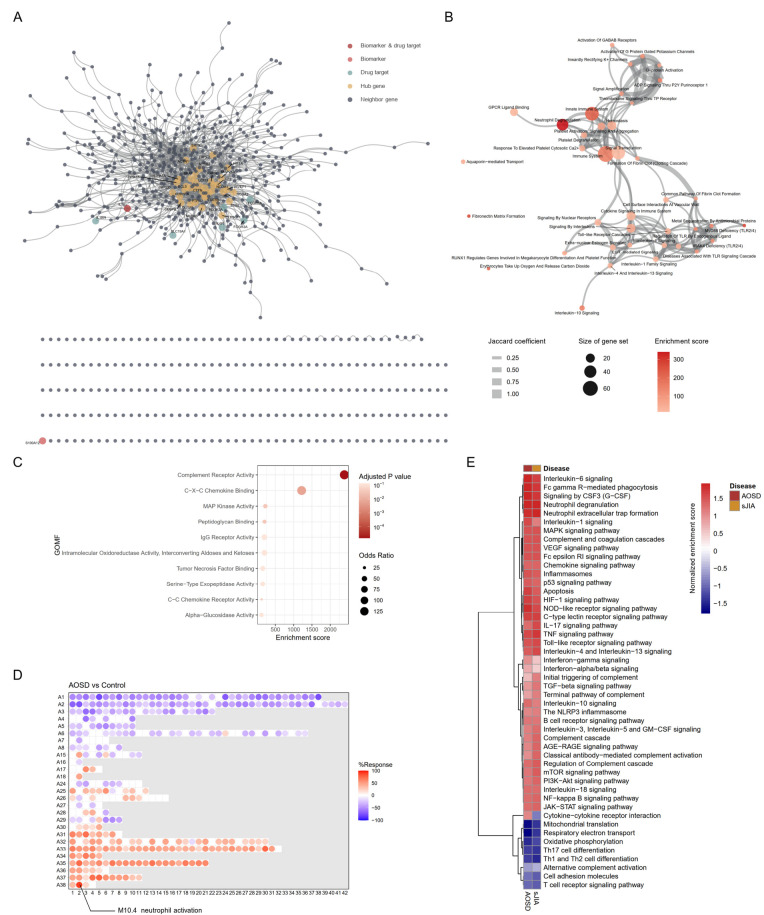
Differentially expressed genes (DEGs) and their functional enrichment. (**A**) DEGs and their functional networks within the human interactome. Biomarkers, drug targets, and hub genes are annotated. (**B**) An enrichment map through a DEG-driven functional enrichment analysis using Enrichr for the Reactome database. (**C**) A functional enrichment analysis using gene sets from Gene Ontology (GO)– Molecular Functions (MF) for 94 DEGs as components of neutrophil degranulation. (**D**) Blood transcriptome modular repertoire analysis. The module response percentage ranges from − 100 to 100, indicated by blue and red, respectively. Detailed information on the modules can be accessed at https://ayllonbe.github.io/modulesV3/index.html. The most responsive module, with a response rate of 88.8%, was M10.4, and it was associated with neutrophil activation. (**E**) Enrichment of AOSD-associated signaling pathways and biological processes in a GSEA. The normalized enrichment score (NES) indicates the relative degree of overrepresentation across the gene sets

In total, 782 upregulated DEGs were identified in blood samples from AOSD patients and 1,472 interactions within the human interactome were detected (Fig. 1A, Supplementary Table 3). The largest connected subnetwork comprised 530 genes. The DEGs included two biomarker genes, IL1B and S100A12, as well as seven drug target molecules: IL1B, FCGR1A, FCGR2A, FCGR3A, IL1RN, IL6R, and SCLC109A1. The primary drug target molecules, including IL1B and IL6R, were integrated into the largest connected subnetwork. In the functional enrichment analysis for gene sets from the Reactome pathway database, the DEGs were most enriched for neutrophil degranulation (Fig. 1B). IL-1 signaling, toll-like receptor cascades, and G-protein-coupled receptor (GPCR) ligand binding were also significantly enriched processes. Ninety-four of the 782 DEGs, accounting for 10.8%, were involved in neutrophil degranulation. These DEGs were significantly enriched for complement receptor activity, C-X-C chemokine binding, MAP kinase activity, peptidoglycan binding, and IgG receptor activity, as determined by functional enrichment analysis using gene sets from GO– Molecular Functions (MF) (Fig. 1C).

To further corroborate the distinct molecular signatures of AOSD, we conducted a blood transcriptome modular repertoire analysis [9, 27], which involved the creation of 382 transcriptional modules based on a collection of reference disease samples. M10.4 was identified as the module most enriched in AOSD (Response = 88.8%) and it was functionally annotated for neutrophil activation (Fig. 1D).

We searched the literature and compiled the biological processes and signaling pathways implicated in the pathogenesis of AOSD [2, 4, 8]. We then performed a GSEA comparing the gene expression profiles of AOSD and sJIA, its pediatric counterpart, with those of healthy controls to understand the differential enrichment of biological processes or pathogenic pathways (Fig. 1E). Most major biological processes and signaling pathways, including neutrophil degranulation, neutrophil extracellular trap formation, signaling by CSF3 (G-CSF), FcγR-mediated phagocytosis, IL-6 signaling, and IL-1 signaling, were comparably enriched in both AOSD and sJIA. However, cytokine-cytokine receptor interactions were activated in AOSD, but not in sJIA. Mitochondria-associated processes, such as mitochondrial translation, respiratory electron transport, oxidative phosphorylation, and the T cell receptor signaling pathway, were suppressed in both AOSD and sJIA.

### Biological pathways associated with neutrophil activation

**Fig. 2:**
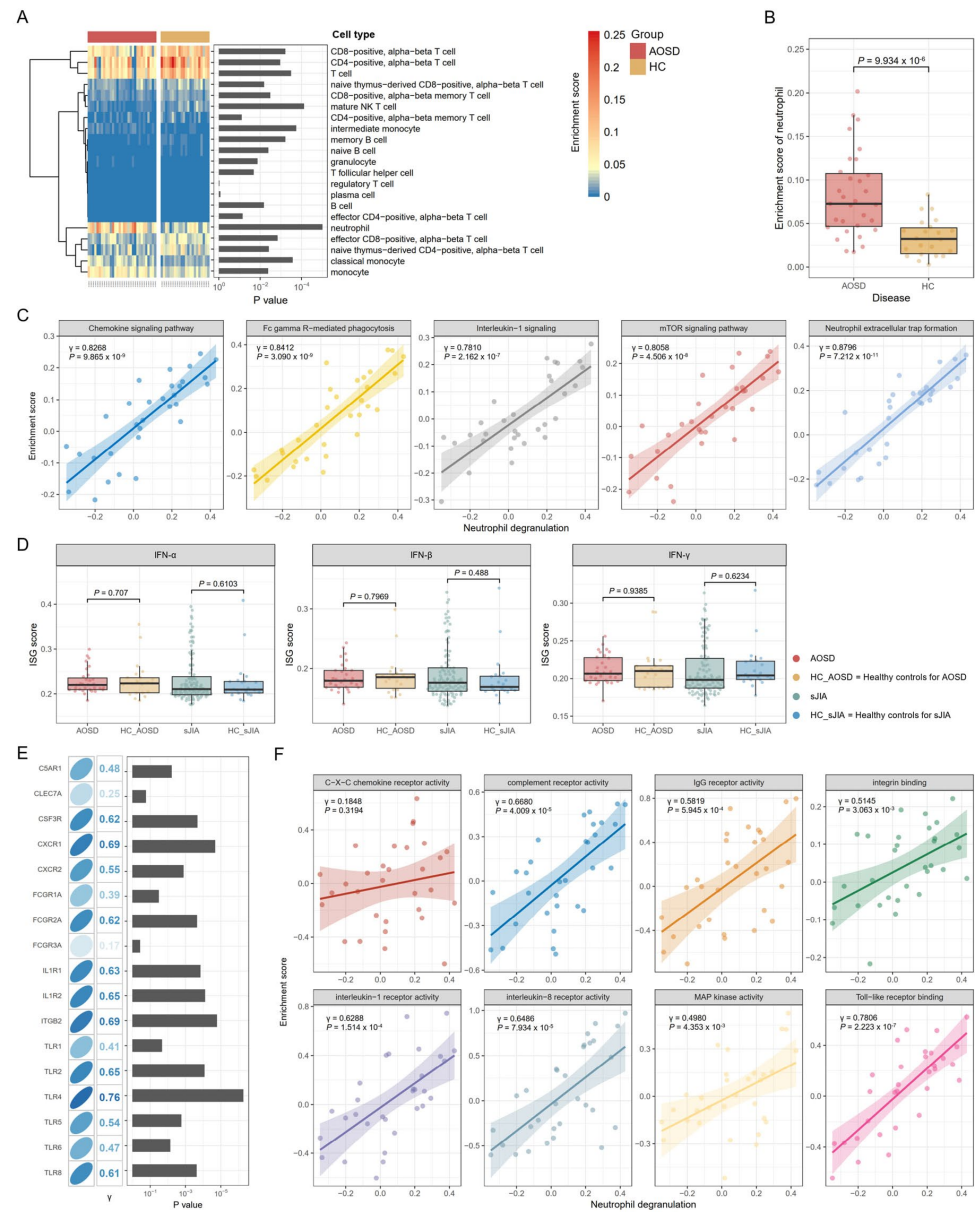
Neutrophil activation and associated biological features. (**A**) Cell type deconvolution analysis using the xCell 2.0 algorithm. (**B**) The enrichment score of neutrophils using the xCell 2.0 algorithm. (**C**) Correlation analysis between neutrophil degranulation and associated pathways or processes. The enrichment score for each individual pathway or process in a single sample was calculated using a gene-set variation analysis (GSVA). (**D**) Interferon-stimulated gene (ISG) scores. IFN = interferon. (**E**) Correlation analysis between neutrophil degranulation and the expression of cell surface receptors that transduce activation signals in neutrophils. (**F**) Correlation analysis between neutrophil degranulation and the activity of molecular functions that transduce activation signals in neutrophils

We estimated cellular enrichment using xCell 2.0, an advanced cell-type deconvolution method [28]. Molecular signatures of neutrophils were most enriched in AOSD, and that enrichment differed most significantly from that of healthy controls (Fig. 2A-B). To determine the differential variation of each biological process or signaling pathway at an individual sample level, we performed a GSVA. In the previous analysis, we confirmed that neutrophil degranulation, a form of neutrophil activation, was the key pathological process in the blood of AOSD patients. Neutrophil degranulation correlated most with neutrophil extracellular trap (NET) formation (γ = 0.8796, *P* = 7.212 × 10^− 11^), followed by FcγR-mediated phagocytosis (γ = 0.8412, *P* = 3.090 × 10^− 9^), the chemokine signaling pathway (γ = 0.8268, *P* = 9.865 × 10^− 9^), the mTOR signaling pathway (γ = 0.8058, *P* = 4.506 × 10^− 8^), and IL-1 signaling (γ = 0.7810, *P* = 2.162 × 10^− 7^) (Fig. 2C).

IFN pathway activation contributes to the development of various autoimmune rheumatic diseases [39, 40]. The involvement of IFNs in the pathogenesis of AOSD has also been reported [41, 42]. We calculated ISG scores across the samples. No significant differences in ISG scores were observed between AOSD patients and healthy controls (Fig. 2D). ISG scores between the sJIA and HC groups were generally comparable; however, a subset of patients with sJIA exhibited elevated ISG scores, as described in the previous study [43]. In AOSD, the type I IFN signaling pathway did not correlate with neutrophil degranulation (γ = 0.2512, *P* = 0.1728), but the type II IFN signaling pathway was moderately correlated (γ = 0.4247, *P* = 0.0172) (Supplementary Fig. 2).

Neutrophils are a significant source of IL-1, which exerts feedback on neutrophils through IL-1 receptors, including IL1R1 and IL1R2 [44]. IL-1-mediated signaling is involved in the migration, survival, and activation of neutrophils [45, 46]. Moreover, GPCRs (CXCR1, CXCR2, C5AR1), Fcγ receptors (FCGR1A, FCGR2A, FCGR3A), β2 integrins (ITGB2), Type I and II cytokine receptors (CSF3R), toll-like receptors (TLR1, TLR2, TLR4, TLR6, TLR8), and C-type lectins (CLEC7A) are the cell surface receptors that transduce intracellular activation signals in neutrophils [47]. These receptors, with the exception of ITGB2 and CLEC7A, were identified as DEGs (Supplementary Table 1). The expression levels of the receptors, excluding CLEC7A and FCGR3A, were significantly positively correlated with neutrophil degranulation (γ = 0.41–0.76, *P* < 0.05) (Fig. 2E).

In a correlative analysis with gene sets from GO-MF, neutrophil degranulation correlated most significantly with toll-like receptor activity (γ = 0.7806, *P* = 2.223 × 10^− 7^), followed by complement receptor activity (γ = 0.6680, *P* = 4.009 × 10^− 5^), IL-8 receptor activity (γ = 0.6486, *P* = 7.934 × 10^− 5^), IL-1 receptor activity (γ = 0.6288, *P* = 1.514 × 10^− 4^), and IgG receptor activity (γ = 0.5819, *P* = 5.945 × 10^− 4^) (Fig. 2F).

### Network-based diffusion effects of target molecules

Genes execute biological functions in collaboration with adjacent genes, proteins, and chemical compounds. Genes situated in proximity within protein-protein interaction (PPI) networks to established disease genes within PPI networks may share biological functions, and thereby possess a greater likelihood of regulating disease modules [48]. By diffusing differential expression signals to neighboring or correlated nodes in the network, genes are prioritized as potential targets based on the transcriptional response of functionally related genes [49]. To determine the potential effects of the genes of interest (GOIs) on key pathological processes, we employed a label propagation and network diffusion algorithm, which models heat flow from the seed genes along interactions in a PPI network [33]. A higher diffusion score for a gene indicated greater likelihood of perturbing the disease module or associated pathway(s).

**Fig. 3:**
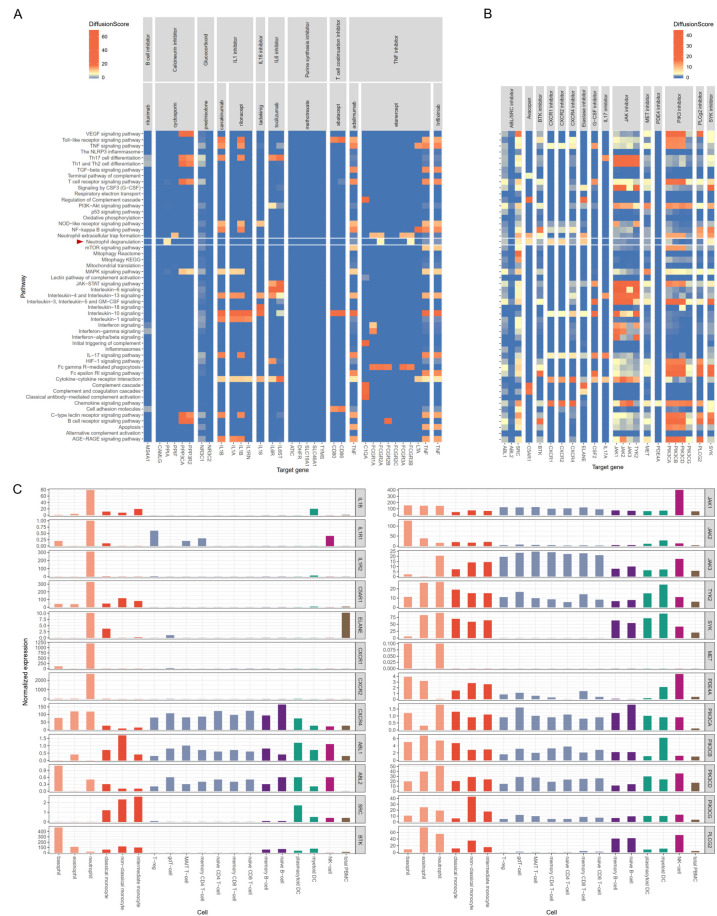
Kernel-based diffusion scores and expression values for drug target molecules. (**A**) Current therapeutic agents for AOSD and their molecular targets and diffusion scores. The diffusion score was calculated using a z-scaled Monte Carlo method for each gene set associated with the pathway. (**B**) Investigational drugs that target neutrophils, their molecular targets, and diffusion scores. (**C**) Expression levels of drug target molecules in subsets of blood immune cells. These data were sourced from the Human Protein Atlas (https://www.proteinatlas.org/)

Among the current treatment options, etanercept and cyclosporine significantly affect neutrophil degranulation. However, that effect occurs through collateral targets (PPIA for cyclosporine, FCGR1A and FCGR3B for etanercept). IL-1 inhibitors (canakinumab and rilonacept), an IL-6 inhibitor (tocilizumab) and an IL-18 inhibitor (tadekinig) did not demonstrate a diffusion score sufficient to influence neutrophil degranulation (Fig. 3A). Several drugs targeting neutrophils are currently under investigation or in clinical trials [47]. In our analyses, ABL/SRC inhibitors, avacopan, CXCR1/CXCR2 inhibitors, neutrophil elastase inhibitors, and SYK inhibitors demonstrated significant diffusion scores for neutrophil degranulation via their target molecules (Fig. 3B).

To evaluate the suitability of the target as an ideal drug target [50], we used the Human Protein Atlas database to analyze the expression levels of genes in blood immune cells [51]. IL1B, IL1R1, IL1R2, C5AR1, ELANE, CXCR1, CXCR2, and MET were predominantly expressed in neutrophils, whereas the other genes exhibited significant expression in various other blood immune cells as well (Fig. 3C). IL1B, C5AR1, and ELANE expression was also observed in monocytes. CXCR1 (fold change = 1.65, *P* = 6.26 × 10^− 4^), CXCR2 (fold change = 1.40, *P* = 4.01 × 10^− 4^), and C5AR1 (fold change = 1.38, *P* = 0.0010) are the genes that satisfy all three of the conditions: they are DEGs, exhibit neutrophil-dominant expression, and have a high diffusion score for neutrophil degranulation. To confirm this result, we imported the single cell RNA sequencing data of human blood cells, including neutrophils, from the GSE137540. C5AR1 (average log_2_FC = 1.397, adjusted *P* value ≈ 0.0), CXCR1 (average log_2_FC = 0.296, adjusted *P* value = 6.59 × 10^− 113^), and CXCR2 (average log_2_FC = 0.423, adjusted *P* value ≈ 0.0) were identified as one of the marker genes for neutrophils and dominantly expressed in neutrophils (Supplementary Fig. 3).

**Fig. 4:**
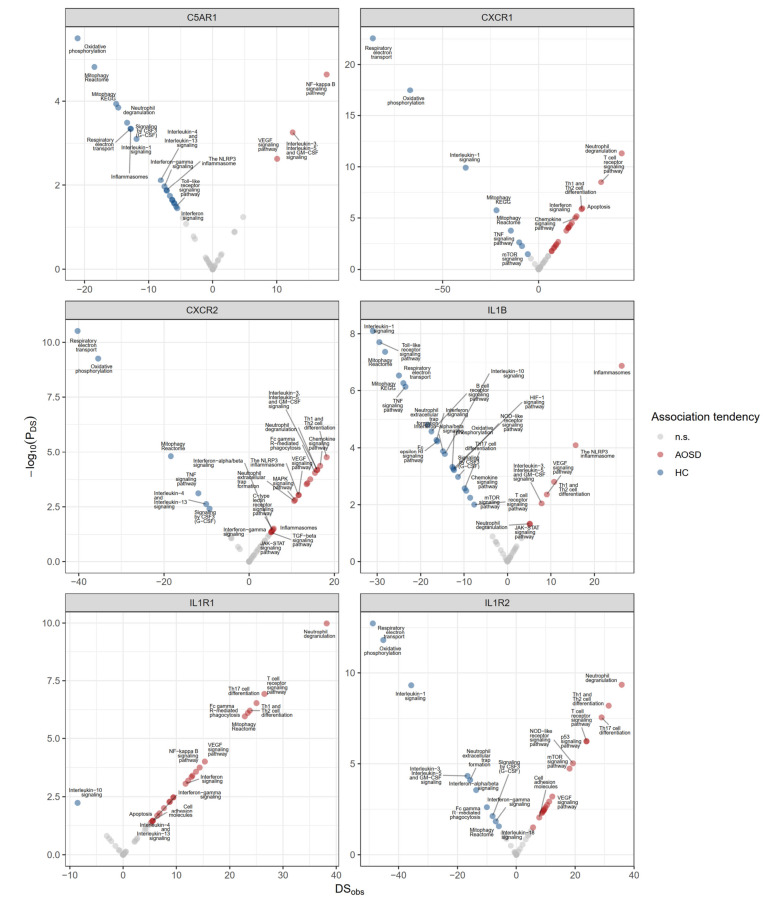
Differential gene-gene set associations enriched in AOSD. Functional associations were detected using the geneCOCOA algorithm [38]. For each gene set, geneCOCOA compares the significance of the association between the gene set (G) and the gene of interest (GOI) under two specified conditions by calculating the ratio of significance values, denoted as the differential score (DS). A DS greater than 0 indicates a stronger association between the GOI and G in the disease condition, and a DS less than 0 suggests stronger associations in the control condition. A p-value, denoted as p(DS), can be derived for each calculated DS

To corroborate the functional associations of the GOI in AOSD, we used a mathematical method to identify the most biologically relevant gene functions within the context of specific gene expression conditions [38]. A differential score (DS) > 0 indicates that the GOI and gene set are more strongly associated in the disease state, and a DS < 0 suggests stronger associations in the healthy condition. In AOSD, CXCR1 and CXCR2 were strongly associated with neutrophil degradation, whereas C5AR1 was linked to the NF-κB and GM-CSF signaling pathways (Fig. 4). IL1R1 and IL1R2 demonstrated a significant association with neutrophil degradation, whereas IL1B exhibited a stronger correlation with inflammasome-related processes (Fig. 4).

## Discussion

For this study, we imported the blood gene expression profiles of AOSD patients and meticulously examined them to elucidate the cellular and molecular features associated with the inflammatory processes of the disease. Furthermore, we identified promising molecular targets that could effectively regulate the key pathological alterations associated with the disease. Neutrophil activation processes, including neutrophil degranulation and the NET formation [52], were significantly stimulated in AOSD patients. Fcγ receptors, IL-1 receptors, and chemokine receptors served as key signals closely associated with neutrophil activation. CXCR1, CXCR2, and C5AR1 were the DEGs predominantly expressed in neutrophils that demonstrated significant potential as target molecules for mitigating neutrophil activation.

Neutrophils are the most abundant leukocytes in human circulation and are considered to be the primary responders to acute inflammatory responses [53, 54]. In AOSD patients, neutrophilia is one of the cardinal findings [1, 2, 4], and the molecular signatures of neutrophils in the blood are most significantly enriched, compared with healthy controls. Because neutrophils are a crucial source of key cytokines, including IL-1, IL-6, CXCL8 (also known as IL-8), and GM-CSF, that mediate the inflammatory response in AOSD [55, 56], they are considered to play a leading role in the inflammatory response, influencing both its magnitude and characteristics. Neutrophils express a variety of activating cell surface receptors that use distinct intracellular signaling mechanisms to mediate their effector functions [47]. In AOSD, the major receptors and their signaling pathways (except for C-type lectin) were identified as activated and closely related to neutrophil activation. Binding to chemokine receptors (CXCR1 and CXCR2) and complement receptor (C5AR1) and their intracellular signaling seem to be particularly specific to neutrophil activation. C5a can be produced by activated neutrophils directly or indirectly, and C5a-induced signaling through C5AR1 is mediated by GPCRs, which ultimately lead to neutrophil degranulation, morphological changes, and chemotaxis [57]. CXCR1 and CXCR2 also use GPCRs, phospholipase Cβ (PLCβ), and phosphoinositide 3-kinase (PI3K) to initiate intracellular signaling, similar to C5AR1 [47, 57]. A close association between GPCRs and neutrophil degranulation was confirmed in DEG-driven enrichment analysis of Reactome pathways (Fig. 1B). In our GSEA analysis in GO-MF, IL-8 receptor activity was found to be more enriched than CXC chemokine receptor activity, and it exhibited a stronger correlation with neutrophil degranulation (Fig. 2F). The gene set for IL-8 receptor activity is defined by only two genes: CXCR1 and CXCR2. In contrast, the gene set for CXC chemokine receptor activity contains eight genes: CXCR1, CXCR2, CXCR3, CXCR4, CXCR5, CXCR6, ACKR3, and GPR35.

IL-1β, along with IL-18, is a well-established cytokine that plays a crucial role in the inflammatory response associated with AOSD. Intense production of IL-1β results from the excessive activation of the NLRP3 inflammasome in macrophages and neutrophils [4]. That activation, in turn, further stimulates these immune cells and contributes to the formation of a cytokine storm by inducing the production of several pro-inflammatory cytokines, including IL-6, IL-8, IL-17, and TNF [4]. Many case reports have provided convincing evidence about the clinical efficacy of agents targeting IL-1β or the IL-1 receptor [4, 8]. But a randomized controlled trial of canakinumab for the treatment of AOSD did not meet its primary endpoint, with only 33% of the canakinumab-treated group achieving remission according to the DAS28-ESR criteria at week 12 [58].

IL-1β is recognized as a crucial activator of neutrophils and a pro-survival cytokine. Its effects might not be independent; rather, they depend on interactions with other cells or soluble molecular factors that prime neutrophils. In an in vitro experiment, IL-1β did not activate highly purified neutrophils or enhance their survival. However, in the presence of peripheral blood mononuclear cells (PBMCs), IL-1β promoted neutrophil survival [59]. Furthermore, an IL-1 receptor antagonist (IL-1ra) did not inhibit lipopolysaccharide- and PBMC-dependent neutrophil survival [59]. Because human circulation contains numerous cellular and soluble factors that transduce activating signals to neutrophils, blocking the interaction between IL-1β and IL-1 receptors might not be sufficient to effectively deactivate neutrophils. In our results, IL-1 inhibitors did not demonstrate a diffusion score favorable for directly degrading neutrophils and NET formation (Fig. 3A). The discrepancy in the context-specific functional association between IL-1β and IL-1 receptors (Fig. 4) suggests that merely blocking additional IL-1β stimulation is insufficient to reverse the ongoing activation process in stimulated neutrophils. In a comprehensive analysis of blood gene expression profiles from canakinumab-treated sJIA patients [13, 43], the activity of the IL-1 signaling pathway itself was not suppressed in correlation with the treatment response. Instead, the treatment response was closely associated with the suppression of neutrophil degranulation. The treatment response to an IL-1 inhibitor could depend on the suppression of neutrophil-driven pro-inflammatory processes through the inhibition of IL-1 signaling pathways, rather than solely on the inhibition of IL-1 itself. Moreover, it is supposed that primed neutrophils do not completely deactivate their inherent mechanisms, even in cases of a clinically complete response, and instead remain primed to reignite. Reports indicate that neutrophils from patients with sJIA who have a long-standing chronic inactive disease status still exhibit elevated expression of inflammatory genes, including components of the inflammasome and S100A8 [60].

Several investigational drugs intended to manipulate neutrophils for the treatment of various inflammatory diseases are currently undergoing clinical trials [47]. We evaluated their therapeutic potential and availability for the treatment of AOSD. Drugs targeting C5AR1, CXCR1, CXCR2, SRC, elastase, and SYK exhibited a diffusion score favorable for neutrophil degranulation. SRC and SYK are intermediate molecules that transfer signals from Fcγ receptors and β2 integrins on the surfaces of neutrophil cells [47]. If the molecule of interest is disease-modifying or has a proven role in the pathophysiology of a disease, and its expression is restricted to the primary cells or tissues responsible for the disease, then the molecule is close to an ideal drug target [50]. C5AR1, CXCR1, and CXCR2 met those criteria because they were among the DEGs of AOSD and are predominantly expressed in neutrophils. CXCR2 antagonism has demonstrated promising results in several chronic inflammatory diseases in which neutrophils play a key role, such as cystic fibrosis, asthma, and chronic obstructive pulmonary disease [47]. Avacopan, an orally available C5a receptor inhibitor, has proven effective in patients with ANCA-associated vasculitis [47]. IL1B, IL1R1, and IL1R2 approximately partially met the aforementioned criteria; however, concerns were raised about their effectiveness in suppressing neutrophil activation.

This study has several limitations that need to be addressed. First, we did not investigate the relationship between molecular features and clinical characteristics (such as fever, skin rash, and arthritis), blood inflammatory markers (including the erythrocyte sedimentation rate, C-reactive protein level, and ferritin level), or the long-term disease course or outcomes due to insufficiently detailed clinical information at the individual level. Second, blood molecular signatures can be influenced by the predominant blood cell subset, likely neutrophils. Neutrophilia is a characteristic finding in the blood of patients with active AOSD. However, distinct molecular signatures have been identified based on the specific characteristics of the disease [9, 61–64]. Furthermore, a more comprehensive single-cell study needed to evaluate single-cell variability. Third, AOSD might have molecular subtypes similar to those found in sJIA [43]. However, we were unable to investigate those molecular subtypes due to the limited sample size. Fourth, the relationship between mRNA and protein expression remains debatable [65]. However, recent studies suggest a correlative relationship between the mRNA and protein levels of CXCR1 and CXCR2 in human neutrophils [66, 67].

Advancing the knowledge of and treatment options for rare diseases such as AOSD faces many challenges that can be mitigated through a comprehensive analysis of gene expression data using advanced bioinformatics techniques, and focusing on efficiency and precision [68]. Neutrophils are the predominant inflammatory cells and contribute to amplified inflammation in AOSD. In this study, we confirmed the pathological significance of neutrophils in the cellular and molecular networks involved in AOSD and have highlighted the key signaling pathways and active mediators involved in neutrophil activation. Furthermore, we identified the potential limitations of current biologic agents and proposed an alternative therapeutic option for more effective regulation of the neutrophil activation involved in the inflammatory processes of AOSD. Agents targeting CXCR1, CXCR2, and C5AR1 are worth considering because clinical trials for several neutrophil-oriented inflammatory diseases are ongoing [47]. This study addresses a significant knowledge gap and promotes the development of promising alternative therapeutic approaches for AOSD.

## Electronic supplementary material

Below is the link to the electronic supplementary material.

Supplementary Material 1: Supplementary Tables 1–2 and Figs. 1, 2 and 3

Supplementary Material 2: Supplementary Table 3

## Data Availability

All raw data are available in a public, open-access repository. The data have been deposited in the Gene Expression Omnibus repository (GEO) under accession number GSE22098 and GSE80060. Metadata and interim processed data are available upon reasonable request from the corresponding author.

## Abbreviations

AOSD: adult-onset Still’s disease
DEGs: differentially expressed genes
DS: differential score
GO: Gene Ontology
GOI: gene of interest
GPCR: G-protein-coupled receptor
GSEA: gene-set enrichment analysis
GSVA: gene-set variation analysis
IFN: interferon
IL: interleukin
ISG: interferon-stimulated gene
KEGG: Kyoto Encyclopedia of Genes and Genomes
MF: Molecular Functions
MSigDB: Molecular Signatures Database
NES: normalized enrichment score
NET: neutrophil extracellular trap
PBMCs: peripheral blood mononuclear cells
sJIA: systemic juvenile idiopathic arthritis
TNF: tumor necrosis factor
