# Supplementary material for: Targeting neutrophil-driven inflammation in adult-onset still’s disease: molecular insights from gene expression profiles
Source: Arthritis Res Ther. 2025 Jul 1;27:130. doi: 10.1186/s13075-025-03598-5 (PMC12210742; doi:10.1186/s13075-025-03598-5)
Supplement: Supplementary file 1 — Supplementary Material 1: Supplementary Tables 1–2 and Figs. 1, 2 and 3 [file 13075_2025_3598_MOESM1_ESM.docx]

**Targeting Neutrophil-driven Inflammation in Adult-onset Still’s Disease: Molecular Insights from Gene Expression Profiles**

In-Woon Baek^1^, Hyoun-Ah Kim^2^, Kyung-Su Park^3^, Ki-Jo Kim^3*^

^1^ Division of Rheumatology, Department of Internal Medicine, Ewha Womans University, College of Medicine, Seoul, Republic of Korea

^2^ Department of Rheumatology, Ajou University School of Medicine, Suwon, Republic of Korea

^3^ Division of Rheumatology, Department of Internal Medicine, St. Vincent’s Hospital, College of Medicine, The Catholic University of Korea, Seoul, Republic of Korea

* Correspondence: md21c@catholic.ac.kr (K-J.K.)

Corresponding author

Ki-Jo Kim, MD., PhD.

St. Vincent’s Hospital, 93 Jungbu-daero, Paldal-gu, Suwon, Gyeonggi-do, 16247, Republic of Korea

Tel: +82-31-249-8156

Fax: +82-31-253-8898

E-mail: md21c@catholic.ac.kr

**Supplementary Tables**

**Dataset descriptions**

(1) **GSE22098**

Berry et al. collected whole blood samples from 12 pediatric *Streptococcus*, 40 pediatric *Staphylococcus*, 31 adult-onset Still’s disease, 82 pediatric systemic lupus erythematosus (SLE) and 28 adult patients with SLE. RNA was extracted, and globin was reduced. The labeled cRNA was hybridized to Illumina Human HT-12 BeadChips. Healthy controls were included to match the patients’ demographic data. These data were utilized to compare blood transcriptomic signatures between major inflammatory and infectious diseases [1, 2].

**Table 1. Demographic characteristics**

| **Patient** | **Age** | **Sex** | **Race** |
| --- | --- | --- | --- |
| Still-1 | 29 | Female | Caucasian |
| Still-2 | 54 | Male | Caucasian |
| Still-3 | 48 | Female | Hispanic |
| Still-4 | 38 | Female | Caucasian |
| Still-5 | 54 | Male | Caucasian |
| Still-6 | 32 | Female | Hispanic |
| Still-7 | 38 | Female | Hispanic |
| Still-8 | 63 | Female | Native American |
| Still-9 | 27 | Male | Caucasian |
| Still-10 | 44 | Female | Caucasian |
| Still-11 | 58 | Female | Caucasian |
| Still-12 | 35 | Female | Asian |
| Still-13 | 70 | Male | Caucasian |
| Still-14 | 31 | Male | Caucasian |
| Still-15 | 64 | Female | Caucasian |
| Still-16 | 42 | Female | Caucasian |
| Still-17 | 51 | Male | Caucasian |
| Still-18 | 25 | Female | Caucasian |
| Still-19 | 19 | Male | Caucasian |
| Still-20 | 26 | Male | Caucasian |
| Still-21 | 59 | Male | Caucasian |
| Still-22 | 83 | Male | Caucasian |
| Still-23 | 22 | Male | Asian |
| Still-24 | 55 | Male | Caucasian |
| Still-25 | 19 | Male | Caucasian |
| Still-26 | 17 | Male | Caucasian |
| Still-27 | 37 | Female | Native American |
| Still-28 | 29 | Female | Caucasian |
| Still-29 | 48 | Female | Hispanic |
| Still-30 | 34 | Male | Caucasian |
| Still-31 | 19 | Male | Caucasian |

Adapted from the paper by Berry at al. 2010 [1].

(2) **GSE80060**

A blood transcriptomic dataset, GSE80060, was constructed from two randomized trials of canakinumab in sJIA [3, 4]. Patients with active sJIA received subcutaneous canakinumab or a placebo, and blood samples for RNA isolation were collected at baseline (n=104) and on day 3 (n=80). Twenty-two samples from matched healthy controls were included in this study. The RNA was extracted and hybridized using Affymetrix microarrays. Details regarding clinical characteristics are presented in the original paper [4].

**Table 2. Demographic and baseline clinical characteristics of the study participants***

| **Characteristics** | **Trial 1** | | **Trial 2,**  **Open-Label Phase** | **Trial 2,**  **Withdrawal Phase** | |
| --- | --- | --- | --- | --- | --- |
|  | **Canakinumab**  **(N=43)** | **Placebo**  **(N=41)** | **Canakinumab**  **(N-177)** | **Canakinumab**  **(N=50)** | **Placebo**  **(N=50)** |
| Sex – no. (%) |  |  |  |  |  |
| Male | 16 (37) | 18 (44) | 79 (45) | 22 (44) | 23 (46) |
| Female | 27 (63) | 23 (56) | 98 (55) | 28 (56) | 27 (54) |
| Race – no. (%)† |  |  |  |  |  |
| White | 40 (93) | 37 (90) | 151 (85) | 41 (82) | 42 (84) |
| Black | 2 (5) | 0 | 7 (4) | 2 (4) | 1 (2) |
| Other | 1 (2) | 4 (10) | 19 (11) | 7 (14) | 7 (14) |
| Age – year |  |  |  |  |  |
| Median | 8.0 | 9.0 | 8.0 | 8.0 | 8.0 |
| Interquartile range | 4.0–13.0 | 6.0–14.0 | 5.0–12.0 | 6.0–12.0 | 5.0–13.0 |
| Body weight – kg |  |  |  |  |  |
| Median | 22.2 | 27.2 | 25.8 | 28.6 | 25.9 |
| Interquartile range | 15.1–47.9 | 21.0–45.5 | 17.8–42.9 | 19.8–44.0 | 18.6–49.4 |
| Disease duration – year |  |  |  |  |  |
| Median | 2.3 | 2.0 | 2.1 | 2.7 | 1.8 |
| Interquartile range | 1.0–4.7 | 1.2–5.2 | 0.8–4.3 | 1.3–6.2 | 0.4–4.3 |
| Use of methotrexate at base – no. (%) | 29 (67) | 24 (59) | 93 (53) | 28 (56) | 26 (52) |
| Prior use of biologic agent – no. (%)‡ | 25 (58) | 23 (56) | 116 (66) | 30 (60) | 27 (54) |
| Anakinra | 16 (37) | 15 (37) | 83 (47) | 25 (50) | 20 (40) |
| Tocilizumab | 1 (2) | 2 (5) | 10 (6) | 4 (8) | 1 (2) |
| Anti-TNF agent or other biologic agents | 14 (33) | 16 (39) | 62 (35) | 14 (28) | 12 (24) |
| Prednisone therapy at baseline – no. (%) | 31 (72) | 28 (68) | 128 (72) | 32 (64) | 30 (60) |
| * No significant differences were observed between the two treatment groups in trials 1 and 2, according to the randomization in the withdrawal phase.  † Race was determined by the investigators.  ‡ A patient could have received one or more biologic agents previously. TNF denotes tumor necrosis factor. | | | | | |

Adapted from the paper by Ruperto at al. 2012 [4].

**Supplementary Figures**


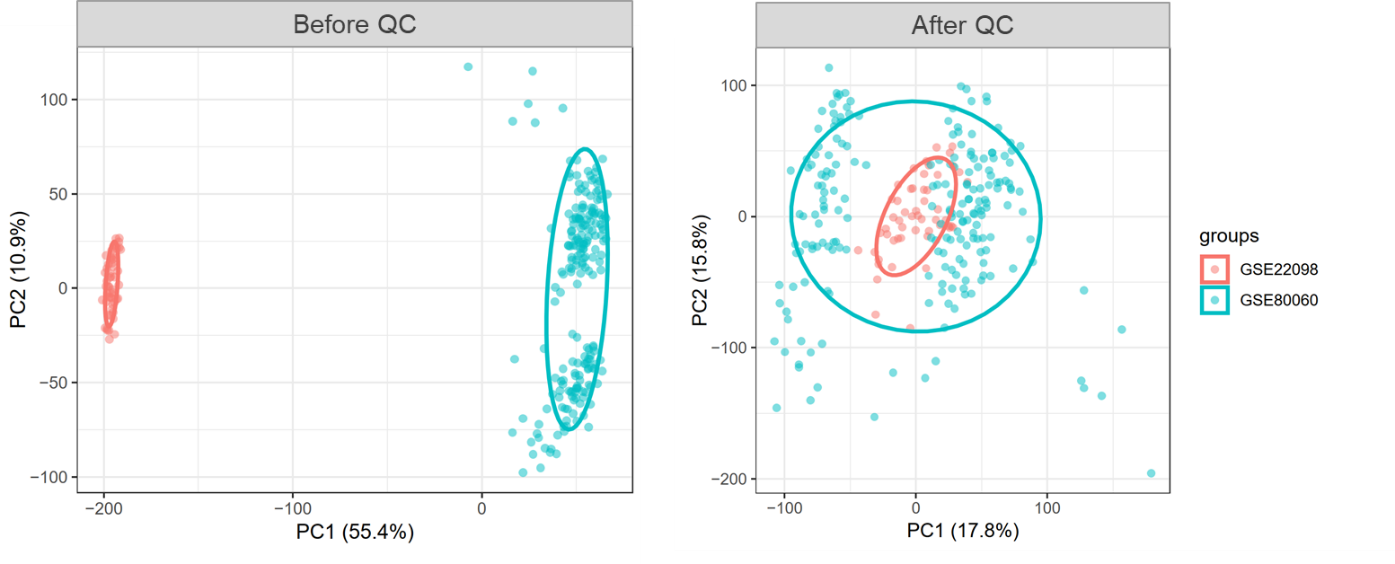


**Figure 1.** Principal component analysis on the integrated datasets of blood transcriptomics before (A) and after (B) normalization and batch correction. QC=quality control.


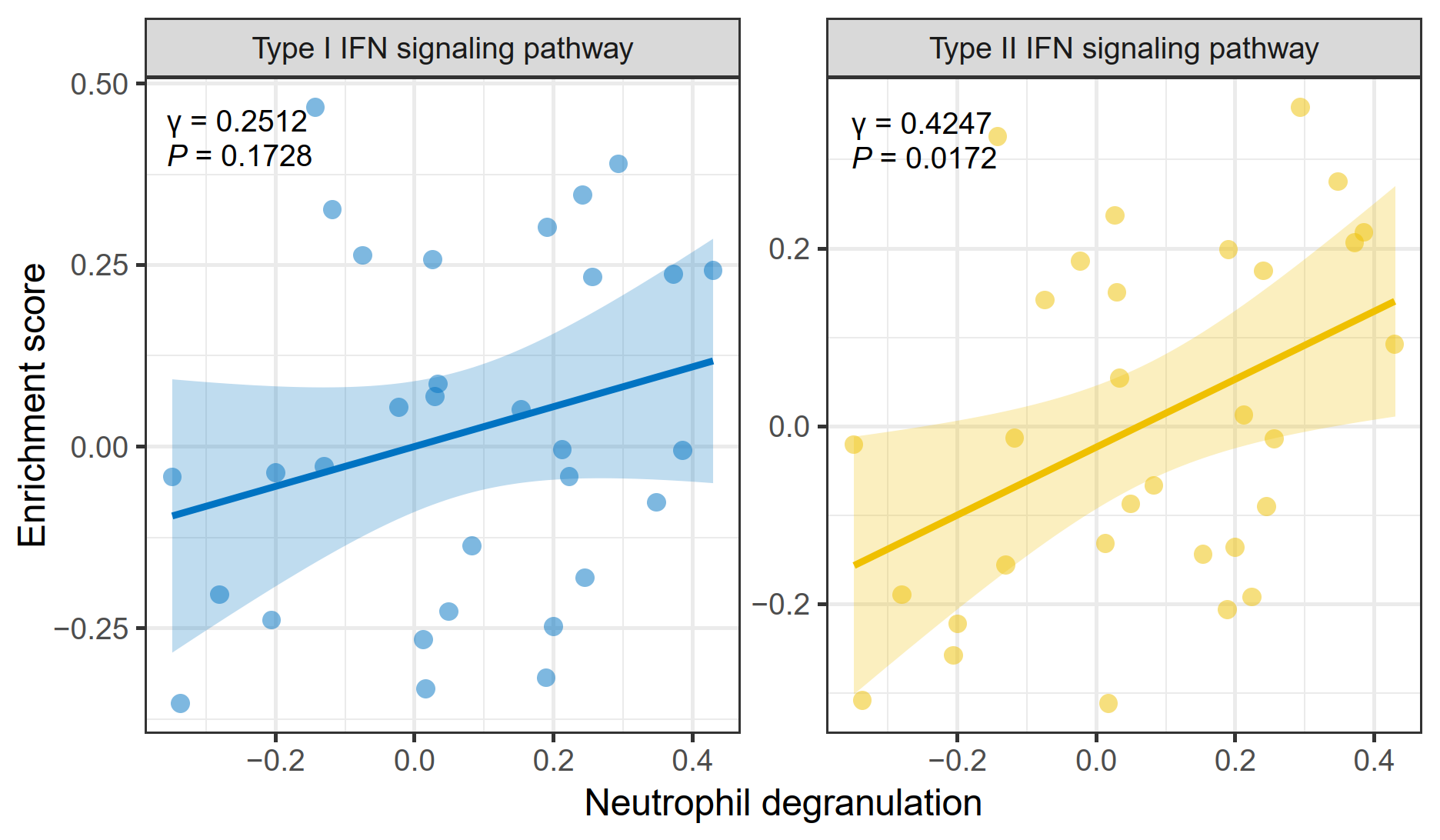


**Figure 2.** Correlation analysis between neutrophil degranulation and interferon (IFN) signaling pathways. The enrichment score for each individual pathway or process in a single sample was calculated using a gene-set variation analysis (GSVA).


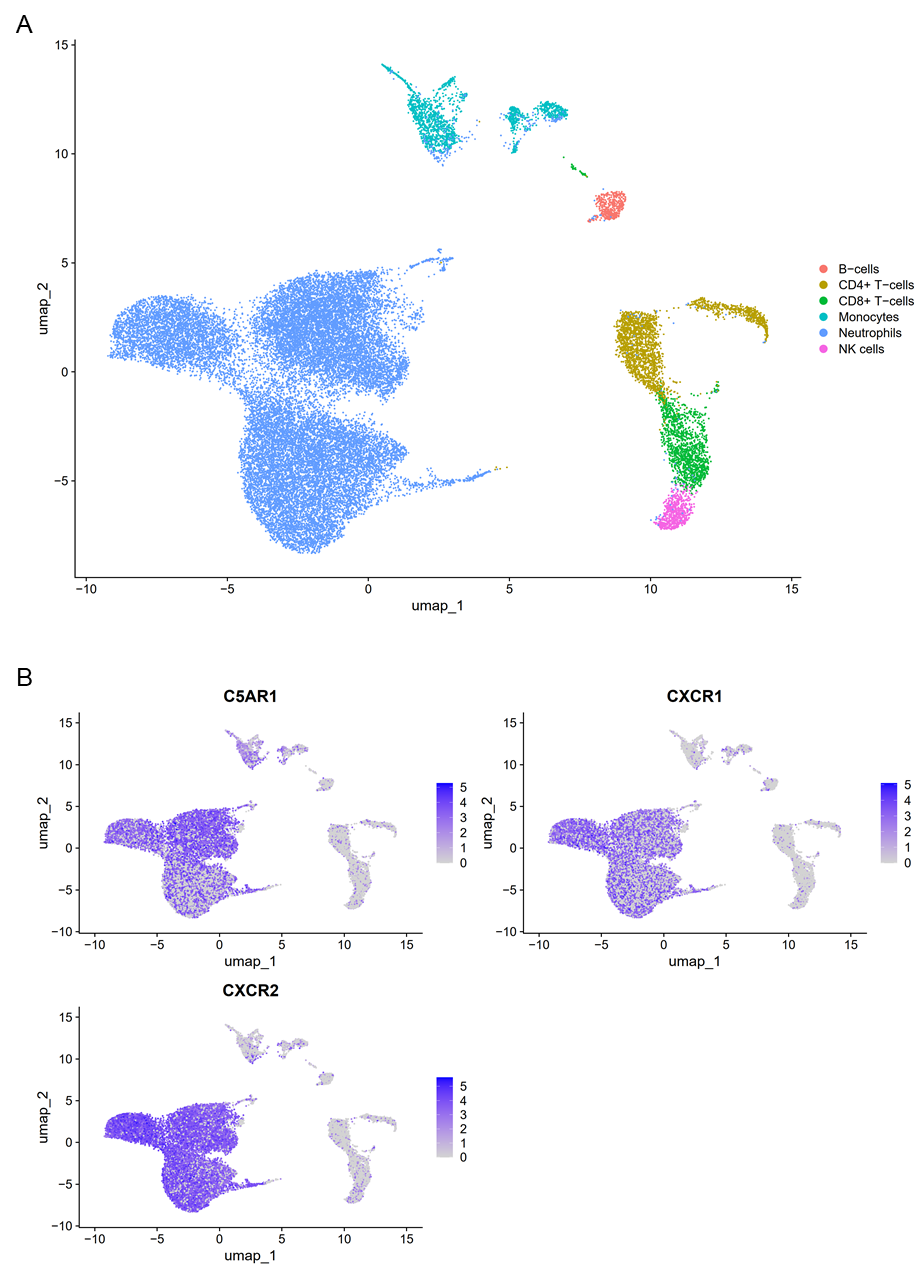


**Figure 3.** Single-cell RNA sequencing of 29,909 single cells isolated from blood of three healthy donors. This data was sourced from GSE137540 [5]. (A) Single-cell uniform manifold approximation and projection (UMAP) plot. Cells are color-coded by computationally determined cell annotations using R package SingleR. (B) Gene expression plots of C5AR1, CXCR1, and CXCR2 across the cell types. Cells expressing the indicated gene are colored purple, and the relative intensity indicates relative expression levels (intensity scale for each plot is on the right).

**References**

1. Berry MP, Graham CM, McNab FW, Xu Z, Bloch SA, Oni T, Wilkinson KA, Banchereau R, Skinner J, Wilkinson RJ *et al*: **An interferon-inducible neutrophil-driven blood transcriptional signature in human tuberculosis**. *Nature* 2010, **466**(7309):973-977.

2. Haynes WA, Haddon DJ, Diep VK, Khatri A, Bongen E, Yiu G, Balboni I, Bolen CR, Mao R, Utz PJ *et al*: **Integrated, multicohort analysis reveals unified signature of systemic lupus erythematosus**. *JCI Insight* 2020, **5**(4):e122312.

3. Brachat AH, Grom AA, Wulffraat N, Brunner HI, Quartier P, Brik R, McCann L, Ozdogan H, Rutkowska-Sak L, Schneider R *et al*: **Early changes in gene expression and inflammatory proteins in systemic juvenile idiopathic arthritis patients on canakinumab therapy**. *Arthritis Res Ther* 2017, **19**(1):13.

4. Ruperto N, Brunner HI, Quartier P, Constantin T, Wulffraat N, Horneff G, Brik R, McCann L, Kasapcopur O, Rutkowska-Sak L *et al*: **Two randomized trials of canakinumab in systemic juvenile idiopathic arthritis**. *N Engl J Med* 2012, **367**(25):2396-2406.

5. Xie X, Shi Q, Wu P, Zhang X, Kambara H, Su J, Yu H, Park SY, Guo R, Ren Q *et al*: **Single-cell transcriptome profiling reveals neutrophil heterogeneity in homeostasis and infection**. *Nat Immunol* 2020, **21**(9):1119-1133.
